# Supplementary figures and images for: Integrative Analysis of Fecal Metagenomics and Metabolomics in Colorectal Cancer
Source: Cancers (Basel). 2020 May 2;12(5):1142. doi: 10.3390/cancers12051142 (PMC7281174; doi:10.3390/cancers12051142)

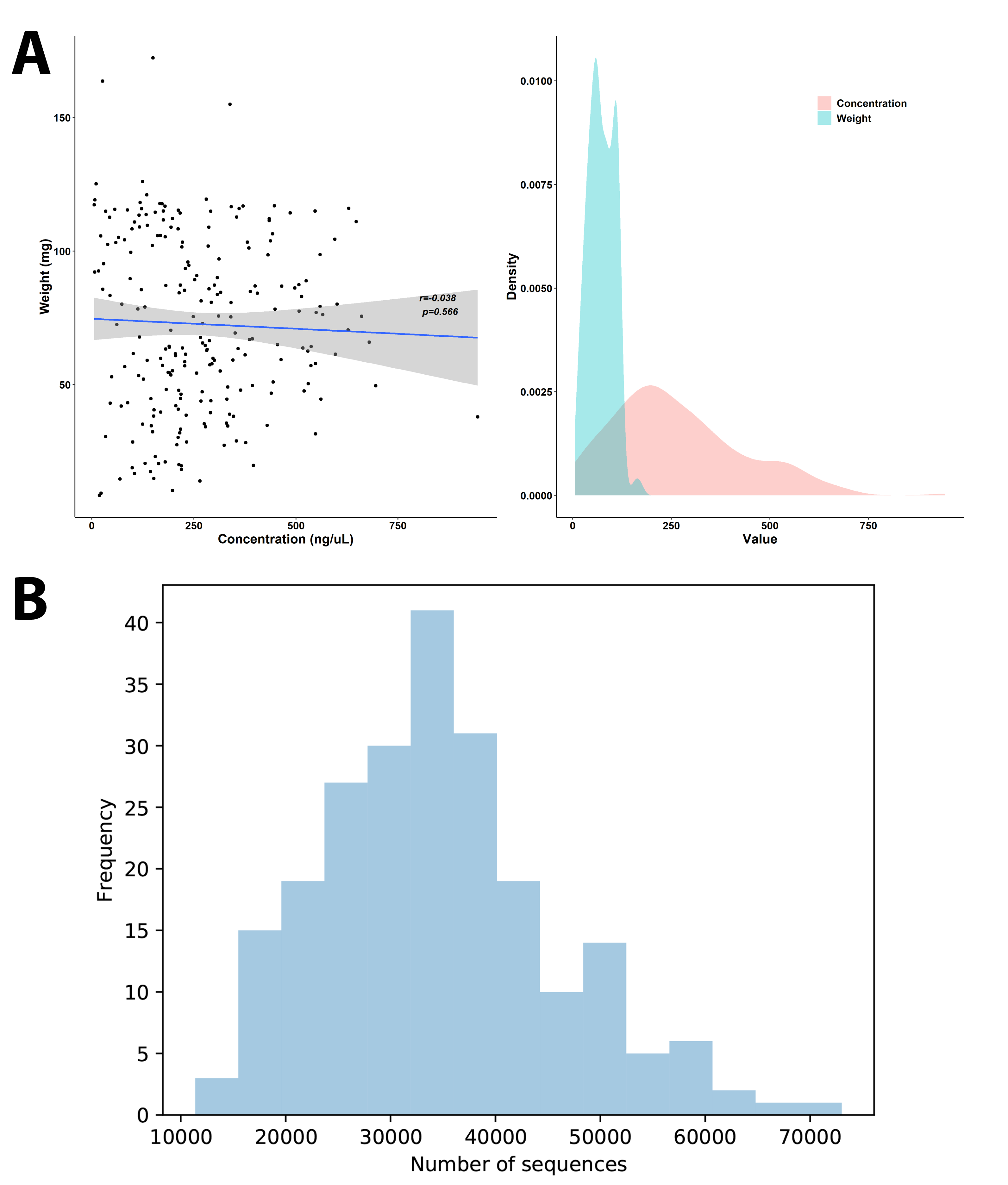

Supplement: Supplementary file 1 [file cancers-12-01142-s001.zip › SUPPLEMENTARY_FIGS1.tif]

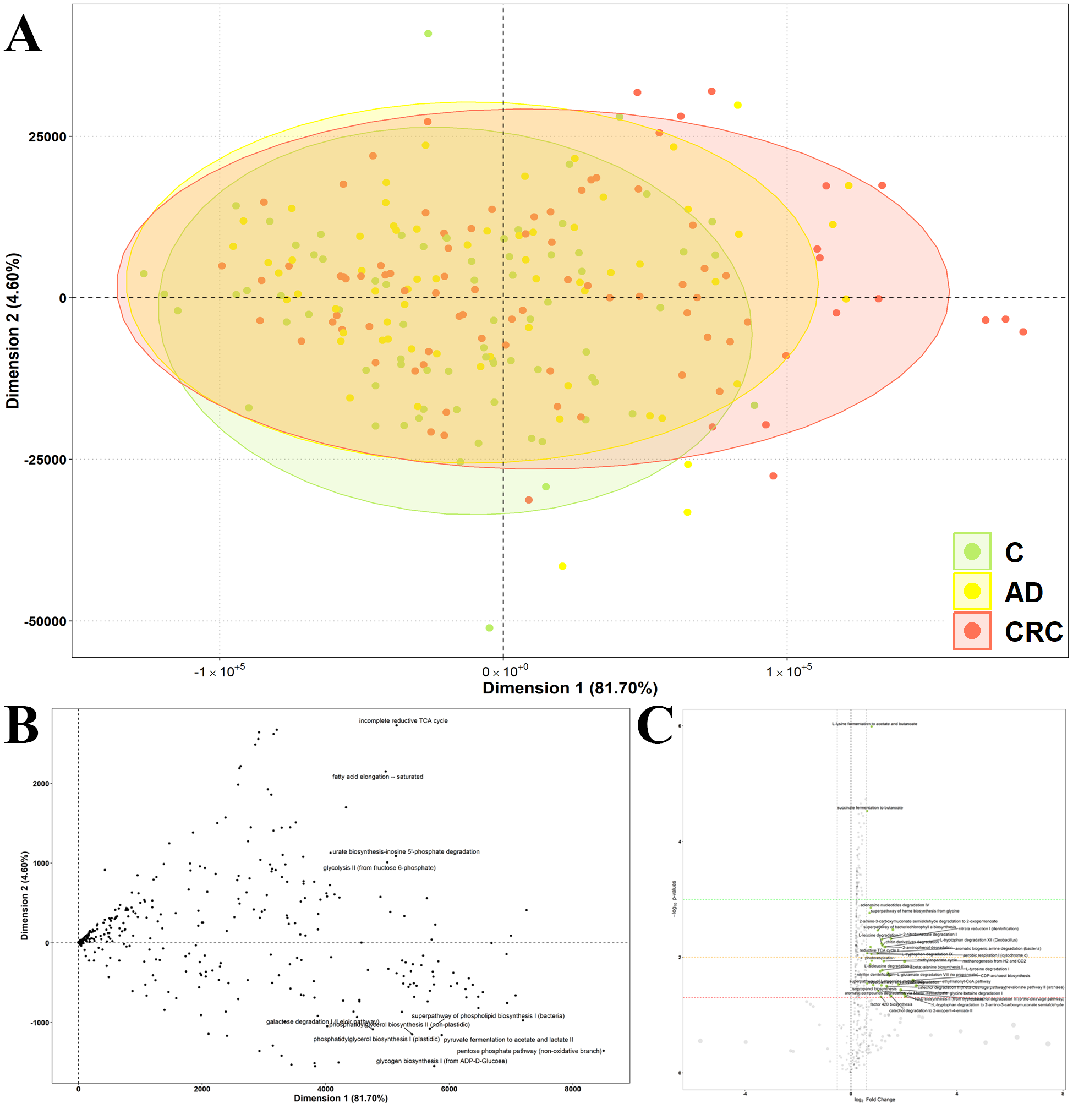

Supplement: Supplementary file 1 [file cancers-12-01142-s001.zip › SUPPLEMENTARY_FIGS3.tif]

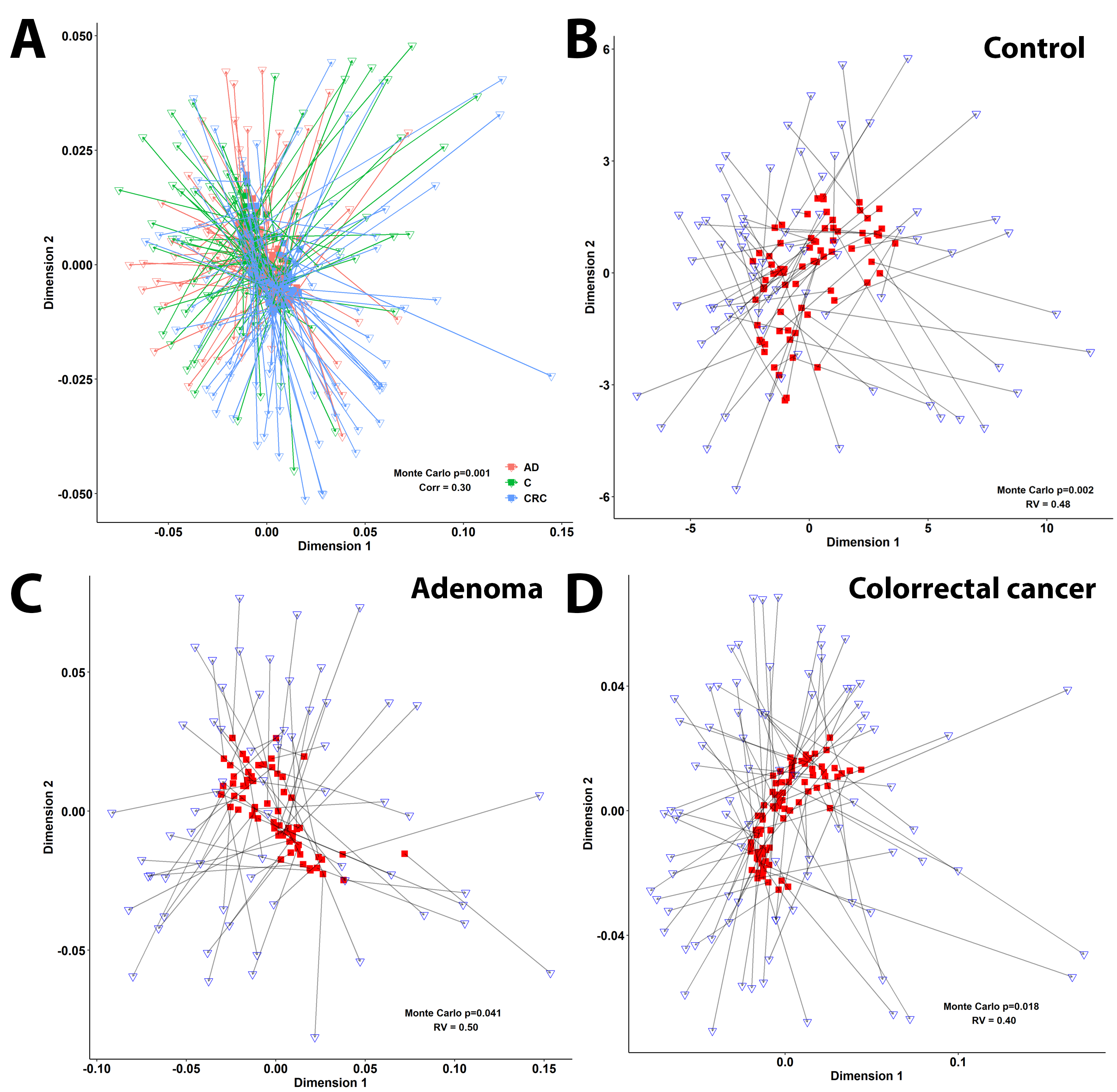

Supplement: Supplementary file 1 [file cancers-12-01142-s001.zip › SUPPLEMENTARY_FIGS5.tif]

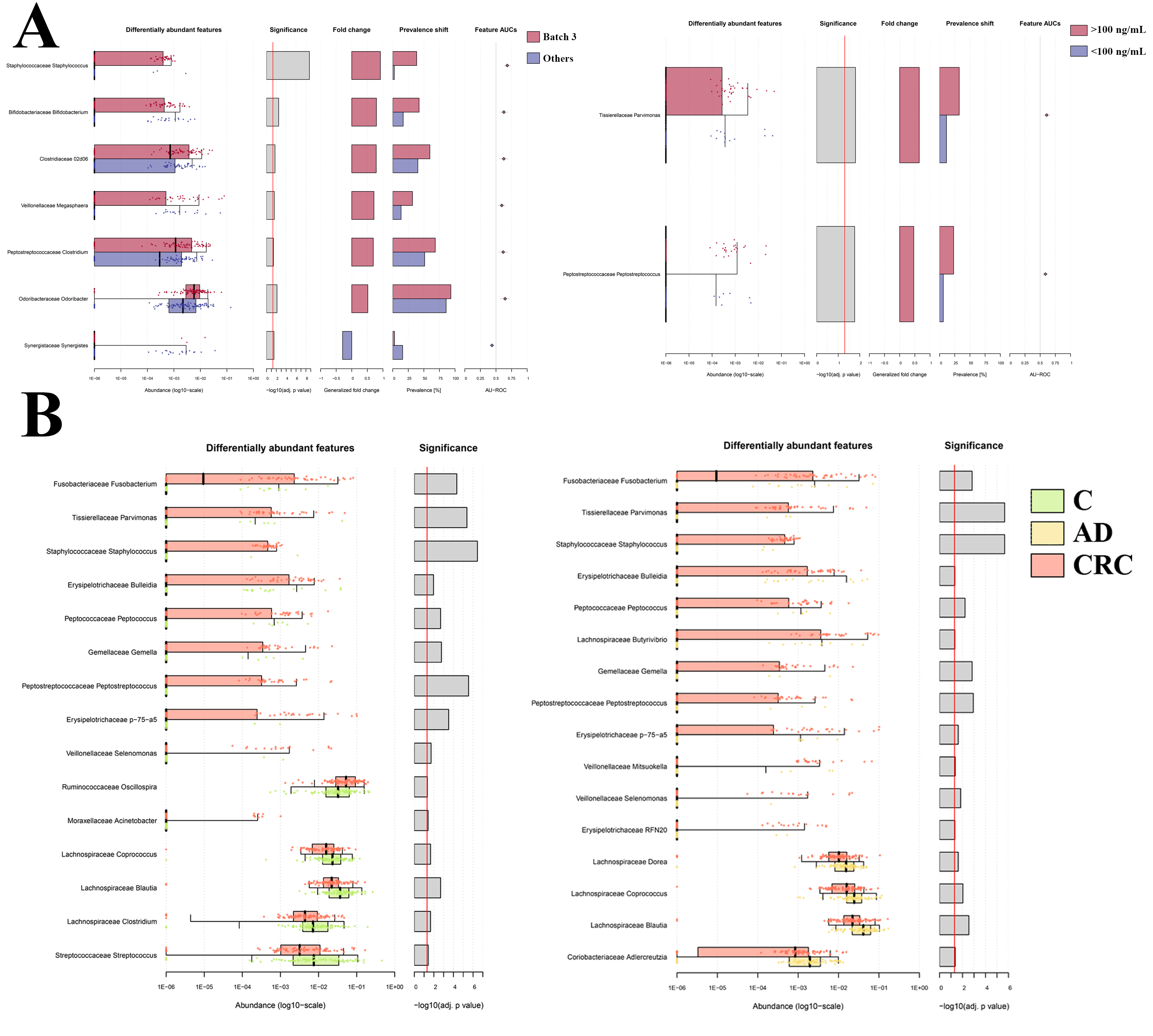

Supplement: Supplementary file 1 [file cancers-12-01142-s001.zip › SUPPLEMENTARY_FIGS2.tif]

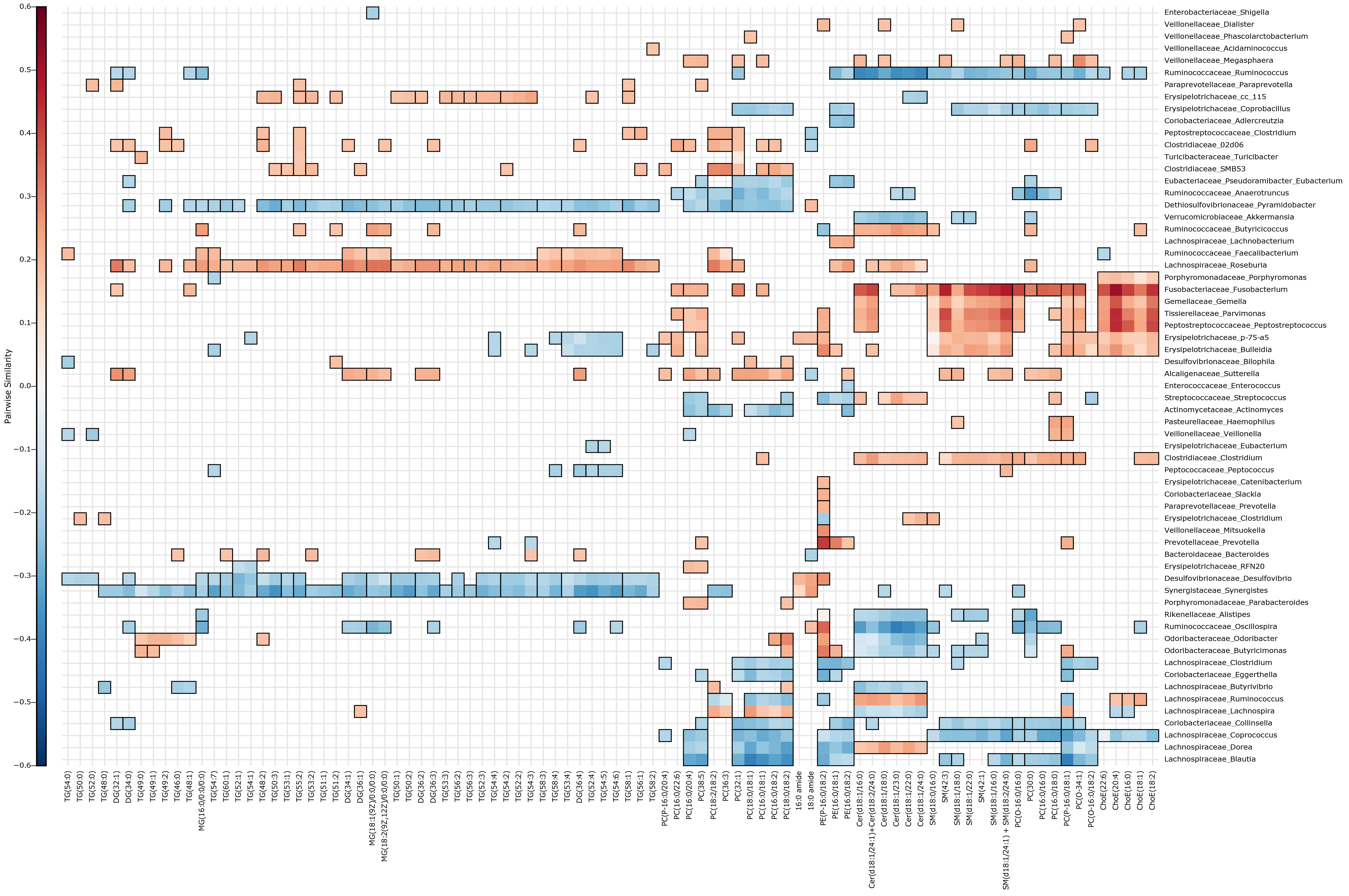

Supplement: Supplementary file 1 [file cancers-12-01142-s001.zip › SUPPLEMENTARY_FIGS4.tif]
